# Current Status of Depression in Patients with Endometriosis and Rheumatoid Arthritis

**DOI:** 10.62641/aep.v53i1.1909

**Published:** 2025-01-05

**Authors:** Shuting Wen, Aiping Zhang, Xin Shi, Junping Hu, Xiaoling Ma, Cheng Peng, Lin Liu, Rongxia He

**Affiliations:** ^1^Reproductive Center, The First Hospital of Lanzhou University, 730000 Lanzhou, Gansu, China; ^2^Department of Obstetrics and Gynecology, Lanzhou University Second Hospital, 730000 Lanzhou, Gansu, China

**Keywords:** depression, endometriosis, immune system diseases, rheumatoid arthritis, impact factors

## Abstract

**Background:**

Endometriosis often causes chronic pain and fertility issues, exacerbating the risk of depression and complicating conditions like rheumatoid arthritis, which further impacts quality of life. This study aimed to explore the detection rate of depression in patients with endometriosis and rheumatoid arthritis by using different diagnostic criteria, and to analyze the occurrence and influencing factors.

**Method:**

A total of 108 patients with endometriosis combined with rheumatoid arthritis in the First Hospital of Lanzhou University from July 2021 to July 2023 were selected as samples. The internationally accepted Diagnostic and Statistical Manual of Mental Disorders, fifth edition (DSM-5), the new depression assessment tool Hamilton Depression Scale (HAMD), and the Self-rating Depression Scale (SDS) were used to detect the incidence of depression in patients with endometriosis and rheumatoid arthritis. On the basis of the DSM-5 results, patients with concurrent depression were categorized into the observation group, and those without depression were categorized into the control group. The patients' clinical data were collected, and the impact factors were analyzed through binary logistic regression.

**Results:**

DSM-5 detected 20 patients with depression, with a detection rate of 18.52%. HAMD detected 21 patients with depression, with a detection rate of 19.44%. SDS detected 18 patients with depression, with a detection rate of 16.67%. The difference in the detection rate of depression in patients with endometriosis combined with rheumatoid arthritis among the three methods was not statistically significant (*p* = 0.865). Binary logistic regression analysis showed that dysmenorrhea (odds ratio (OR) = 3.589, *p* = 0.005), dyspareunia (OR = 2.964, *p* = 0.012), Visual Analog Scale score (OR = 2.545, *p* = 0.001), Disease Activity Score-28 score (OR = 3.828, *p* = 0.004), Pittsburgh Sleep Quality Index score (OR = 3.942, *p* = 0.004), and Health Assessment Questionnaire-Disability Index score (OR = 3.527, *p* = 0.008) were significant influencing factors for depression.

**Conclusion:**

DSM-5, HAMD, and SDS can be used to detect depression in patients with endometriosis and rheumatoid arthritis as effective tools for depression screening. Dysmenorrhea, dyspareunia, Visual Analog Scale (VAS), Rheumatoid arthritis disease activity (DAS28), Pittsburgh Sleep Quality Index (PSQI), and Health Assessment Questionnaire-Disability Index (HAQ-DI) are influencing factors of depression in these patients.

## Introduction

Endometriosis is a common gynecological disease [1, 2]. The main symptoms are chronic pelvic pain, dysmenorrhea, and sexual intercourse pain. It affects female fertility, leads to infertility, menstrual disorders, and other problems, and increases the risk of ectopic pregnancy [3, 4, 5]. The depression in endometriosis group is believed to be underestimated. Another study [6] showed that 15.1% of women with endometriosis were diagnosed with depression. A cross-sectional study by Bernarda Škegro *et al*. [7] showed that 44.3% of patients with endometriosis had depressive symptoms.

Patients with endometriosis are often complicated by immune system diseases [8, 9], which are complicated by rheumatoid arthritis, hypothyroidism, allergic asthma, multiple sclerosis, systemic lupus erythematosus, Crohn’s disease, and ulcerative colitis. The probability of immune system diseases, such as colitis, is significantly increased [10, 11]. A large-scale cohort study conducted by Shih-Fen Chen *et al*. [12] revealed that patients with endometriosis have an increased risk of rheumatoid arthritis (Hazard Ratio (HR): 3.71, 95% confidence interval (CI): 2.91–5.73). As a previous study has shown, rheumatoid arthritis can increase the risk of depression. A cross-sectional analysis of 156 patients with rheumatoid arthritis showed that the prevalence of depression in patients with rheumatoid arthritis (RA) was 76.3%. The majority of patients (49.4%) suffered from moderate-to-severe depression, 91% experienced sleep disorder symptoms, and 21.8% reported negative thoughts of suicidal ideation or self-harm [13].

Secondary depression seriously affects the life quality of patients with endometriosis [14]. Arthritis further reduces patients’ quality of life and prognosis, so identifying risk factors for depression in people with comorbid endometriosis and rheumatoid arthritis is necessary. However, no relevant studies have been found. For this special population, the sensitivity of different depression screening scales is worth exploring. Therefore, a retrospective research was conducted to explore the role of depression in endometriosis when using different diagnostic criteria and scoring scales. The detection rate in patients with endometriosis and rheumatoid arthritis was investigated, and the influencing factors of depression were analyzed.

## Materials and Methods

### General Information

A total of 119 patients with endometriosis complicated with rheumatoid arthritis diagnosed and treated in the First Hospital of Lanzhou University from July 2021 to July 2023 were selected as research subjects, and complete information of 108 patients was received for research. This research has been approved by the Ethics Committee of the First Hospital of Lanzhou University and obtained an ethics certificate (LDYYSZLLKH2024-06). Based on the principle of confidentiality, the personal and family information of patients with endometriosis and rheumatoid arthritis were strictly kept confidential. Informed consent was obtained from all participants. This study adhered to the principles outlined in the Declaration of Helsinki.

### Inclusion and Exclusion Criteria

The inclusion criteria were as follows: (1) Patients aged ≥18 years old; (2) patients diagnosed with endometriosis; (3) patients diagnosed with rheumatoid arthritis; (4) patients without hearing, intelligence, nor language communication impairment and can communicate with others and medical staff; (5) patients who gave informed consent.

The exclusion criteria were as follows: (1) patients joining other clinical trials; (2) patients who have taken psychotropic drugs 2 weeks before admission; (3) patients with schizophrenia, bipolar disorder, paranoid disorder, and other serious mental illnesses; (4) patients with adenomyosis and other diseases that cause pelvic pain and infertility; (5) patients with acute attacks of vaginal bleeding, fever, infection, etc.; (6) patients with developmental malformations of reproductive organs; (7) patients with tumors or serious diseases of other organs.

### Method

All 108 patients were tested for depression 10 minutes after arriving at the diagnosis and treatment site, using the internationally accepted Diagnostic and Statistical Manual of Mental Disorders, fifth edition (DSM-5) [15], the new depression assessment tool Hamilton Depression Scale (HAMD) [16], and the Self-rating Depression Scale (SDS) [17] to detect the incidence of depression in patients with endometriosis and rheumatoid arthritis.

The DSM-5 diagnostic criteria for depression are as follows: persistent low mood; slow thinking and association; inhibition of volition and behavior; decreased interests and hobbies; low self-evaluation, accompanied by insomnia and early awakening, loss of appetite, and decreased sexual desire; and repeated thoughts of death or self-injury or self-abandonment behavior lasting for more than 2 weeks (severe case). Depression triggered by organic brain diseases, physical diseases, and other neuroses like anxiety disorders and obsessive-compulsive disorder is ruled out. In this study, the DSM-5 assessments were conducted by clinicians who had received systematic training in DSM-5 standards. All evaluators underwent comprehensive DSM-5 training and adhered strictly to standardized procedures during the evaluation process.

HAMD has 17 items in total. It uses a five-level scoring method, with scores ranging from 0 to 4. A total score of more than 24 is considered severe depression, more than 17 is considered mild-to-moderate depression, and a score less than 7 is considered to have no depressive symptoms. The HAMD’s reliability is 0.845, its validity is 0.926, and its Cronbach’s alpha coefficient is 0.856.

SDS has a total of 20 items, including 10 items for forward testing and 10 items for reverse testing. It adopts a four-level scoring method and is assigned 1–4 points. The scores of all items are added up and multiplied by 1.25 to obtain the total score (integer is taken), and ≥53 is classified as depressive state. The SDS’s reliability is 0.884, its validity is 0.906, and its Cronbach’s alpha coefficient is 0.931.

On the basis of DSM-5 detection results, patients with endometriosis and rheumatoid arthritis who were complicated by depression were categorized into observation group, and those with endometriosis and rheumatoid arthritis without depression were categorized into control group.

### Evaluation Criteria

(1) The internationally accepted DSM-5 depression diagnostic criteria and new depression assessment tools HAMD and SDS were used to detect the occurrence of depression in patients with endometriosis and rheumatoid arthritis.

(2) The patients’ general demographic information, including age, Body Mass Index (BMI), family history, comorbidities (diabetes, hypertension, hyperlipidemia, and coronary heart disease), educational level, working status, marital status, childbirth history, whether smoking or not, and whether drinking or not, regular exercise habits, and average sleep duration, were collected. The staging of endometriosis according to the American Society for Reproductive Medicine (ASRM). The ASRM classification system is divided into four stages or grades according to the number of lesions and depth of infiltration: minimal (Stage I), mild (Stage II), moderate (Stage III) and severe (Stage IV) [18].

(3) The clinical symptom data of patients, including dysmenorrhea, dyspareunia, pelvic pain, painful defecation, painful urination, and infertility, were collected. The Visual Analog Scale (VAS) score range is 0–10, with 0 and 10 points representing painless and unbearable severe pain states, respectively. The obtained score is directly proportional to the patient’s pain level. The VAS reliability is 0.950, its validity is 0.803, and its Cronbach’s alpha coefficient is 0.865. Rheumatoid arthritis disease activity (DAS28), DAS28 <2.6 points for disease remission, 2.6–<3.2 is classified as low disease activity, 3.2–5.1 is classified as medium disease activity, and >5.1 is classified as high disease activity. The higher the score, the more severe the disease activity. The Pittsburgh Sleep Quality Index (PSQI) score ranges from 0 point to 21 points. The higher the score, the worse the sleep quality. PSQI’s reliability is 0.994, its validity is 0.824, and its Cronbach’s alpha coefficient is 0.845. The Health Assessment Questionnaire-Disability Index (HAQ-DI) was applied to assess the functional status of patients with rheumatoid arthritis. The patients answered 20 questions involving eight functional aspects (dressing, getting up, eating, walking, personal hygiene, touching objects, pinching objects, and activities). Select and score on 4 levels (0 to 3 points). The higher the score, the more severe the physical function limitation is. The average of the eight functional dimension scores is the total HAQ-DI score, with 0 point indicating no functional limitation, 0 points < a score of ≤ 1 defined as mild functional limitation, 1 < score ≤ 2 classified as moderate functional limitation, and 2 < score ≤ 3 classified as severe functional limitation. The test-retest reliability of HAQ-DI is 0.84, and the internal consistency is 0.86, indicating good reliability and validity among the Chinese population with rheumatoid arthritis.

### Statistical Methods

SPSS software version 21.0 (IBM Corporation, Armonk, NY, USA) was used for statistical analysis. This study used the Shapiro-Wilk test to assess the normality of continuous variables. Measurement indicators that conformed to normal distribution, such as age and BMI, were recorded as mean ± standard deviation. Comparisons between groups were processed by independent sample *t *tests. Counting indicators, such as family history and comorbidities, were recorded using [number of cases (percent)] records. Comparison between groups was performed using χ^2^ test, when the theoretical frequency T ≥5 and the sample size N ≥40, use the chi-square test; when 1 ≤ T < 5 and N ≥40, use the continuity correction chi-square test; when T <1 or N <40, use Fisher’s exact test. The influencing factors of depression in patients with endometriosis and rheumatoid arthritis were analyzed using logistic regression. *p*-value < 0.05 was considered statistically significant.

## Results

### Detection of the Incidence of Depression in Patients with Endometriosis and Rheumatoid Arthritis

**Fig. 1.**
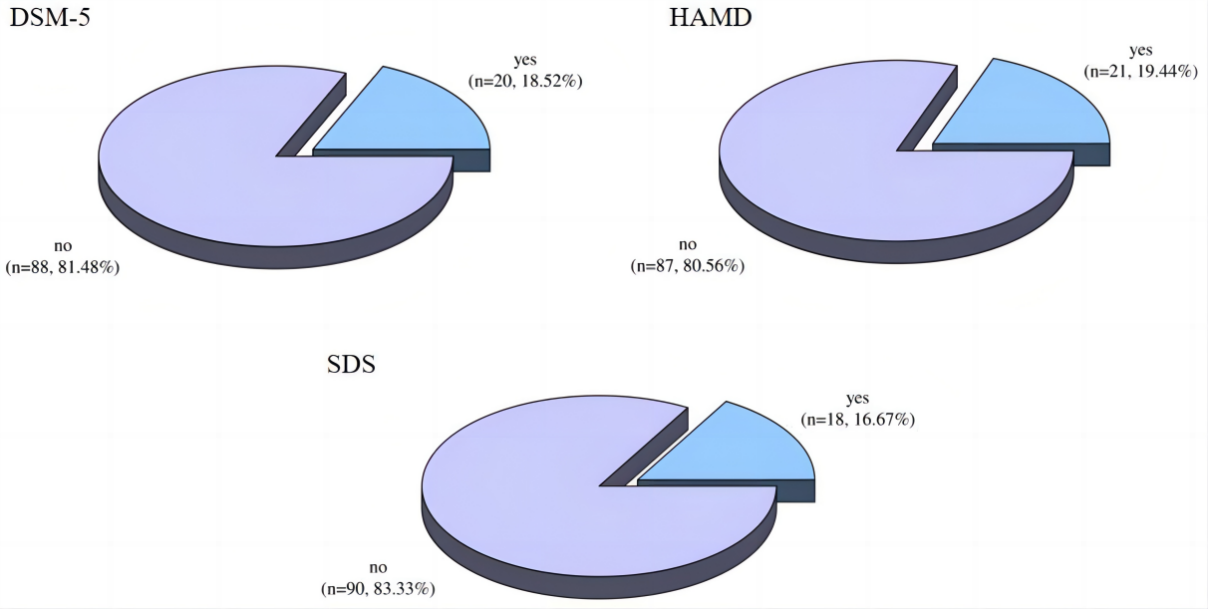
**Detection of depression in patients with endometriosis and rheumatoid arthritis**. DSM-5, Diagnostic and Statistical Manual of Mental Disorders, fifth edition; HAMD, Hamilton Depression Scale; SDS, Self-rating Depression Scale.

DSM-5, HAMD, and SDS were used to detect the incidence of depression in patients with endometriosis and rheumatoid arthritis. DSM-5 detected 20 patients with depression, with a detection rate of 18.52%; HAMD detected 21 patients with depression, with a detection rate of 19.44%; and SDS detected 18 patients with depression, with a detection rate of 16.67% (Fig. 1). No significant difference was found in the detection rate of depression in patients with endometriosis combined with rheumatoid arthritis among the three methods (χ^2^ = 0.290, *p* = 0.865).

### Comparison of General Demographic Information between the Two Groups of Patients

In terms of BMI, family history, diabetes, hypertension, hyperlipidemia, coronary heart disease, working status, marital status, whether smoking, whether drinking alcohol, and regular exercise habits between the observation group and the control group (*p* > 0.05). The patients in the observation group were younger, and they had a higher proportion of high school and below, a higher proportion of childless children, and shorter average sleep time than those in the control group, and the differences were statistically significant (*p* < 0.001, Table 1).

**Table 1.** **Comparison of general demographic information of the two groups of patients**.

General demographic information		Observation group (n = 20)	Control group (n = 88)	χ^2^/*t*	*p*
Age (years old)		37.15 ± 7.36	47.17 ± 6.87	5.813	<0.001
BMI (kg/m^2^)		20.25 ± 0.68	20.33 ± 0.72	0.459	0.647
Family history (n, %)				0.000	1.000*
	Have	3 (15.00%)	12 (13.64%)		
	None	17 (85.00%)	76 (86.36%)		
Diabetes (n, %)				0.000	1.000*
	Have	5 (25.00%)	20 (22.73%)		
	None	15 (75.00%)	68 (77.27%)		
Hypertension (n, %)				0.000	1.000*
	Have	4 (20.00%)	18 (20.45%)		
	None	16 (80.00%)	70 (79.55%)		
Hyperlipidemia (n, %)				0.000	1.000*
	Have	3 (15.00%)	15 (17.05%)		
	None	17 (85.00%)	73 (82.95%)		
Coronary heart disease (n, %)				0.040	0.842*
	Have	2 (10.00%)	13 (14.77%)		
	None	18 (90.00%)	75 (85.23%)		
Educational level (n, %)				15.621	<0.001*
	High school and below	11 (55.00%)	11 (12.50%)		
	College degree and above	9 (45.00%)	77 (87.50%)		
Working status (n, %)				0.000	1.000*
	Employment	17 (85.00%)	73 (82.95%)		
	Not employed	3 (15.00%)	15 (17.05%)		
Marital status (n, %)				—	0.185**
	Unmarried	3 (15.00%)	6 (6.82%)		
	Married	14 (70.00%)	76 (86.36%)		
	Divorced	2 (10.00%)	4 (4.55%)		
	Widowed	1 (5.00%)	2 (2.27%)		
Reproductive history (n, %)				19.514	<0.001
	Sterile	13 (65.00%)	15 (17.05%)		
	Already bred	7 (35.00%)	73 (82.95%)		
Smoking or not (n, %)				0.020	0.887
	Yes	6 (30.00%)	25 (28.41%)		
	No	14 (70.00%)	63 (71.59%)		
Drinking alcohol or not (n, %)				0.000	1.000*
	Yes	5 (25.00%)	20 (22.73%)		
	No	15 (75.00%)	68 (77.27%)		
Has a habit of regular exercise (n, %)				0.345	0.557
	Yes	9 (45.00%)	46 (52.27%)		
	No	11 (55.00%)	42 (47.73%)		
Average sleep time (h)		6.50 ± 0.61	7.66 ± 0.57	8.170	<0.001
Stages of endometriosis				—	0.777**
	Phase 1	2 (10.00%)	7 (7.95%)		
	Phase II	4 (20.00%)	26 (29.55%)		
	Phase III	7 (35.00%)	31 (35.23%)		
	Phase IV	7 (35.00%)	24 (27.27%)		

Note: The staging of endometriosis according to the American Society for Reproductive Medicine; *, the continuity correction chi-square test was used; **, Fisher’s exact test was used. BMI, Body Mass Index.

### Comparison of Clinical Symptom Data between the Two Groups of Patients

No statistically significant difference was found between the observation group and the control group in terms of clinical symptoms such as painful defecation, painful urination, and infertility (*p* > 0.05). The observation group had a higher proportion of dysmenorrhea (*p *= 0.002), dyspareunia (*p *= 0.002), and pelvic pain (*p *= 0.004) and higher scores of VAS, DAS28, PSQI, and HAQ-DI than the control group (*p* < 0.001, Table 2).

**Table 2.** **Comparison of clinical symptoms data between the two groups of patients**.

Clinical symptom information		Observation group (n = 20)	Control group (n = 88)	χ^2^/*t*	*p*
Dysmenorrhea (n, %)				9.281	0.002
	Yes	15 (75.00%)	33 (37.50%)		
	No	5 (25.00%)	55 (62.50%)		
Dyspareunia (n, %)				9.255	0.002
	Yes	12 (60.00%)	22 (25.00%)		
	No	8 (40.00%)	66 (75.00%)		
Pelvic pain (n, %)				8.294	0.004
	Yes	11 (55.00%)	20 (22.73%)		
	No	9 (45.00%)	68 (77.27%)		
Painful defecation (n, %)				1.207	0.272*
	Yes	7 (35.00%)	18 (20.45%)		
	No	13 (65.00%)	70 (79.55%)		
Painful urination (n, %)				0.258	0.612*
	Yes	5 (25.00%)	15 (17.05%)		
	No	15 (75.00%)	73 (82.95%)		
Infertility (n, %)				0.601	0.438*
	Yes	5 (25.00%)	13 (14.77%)		
	No	15 (75.00%)	75 (85.23%)		
VAS (points)		8.05 ± 1.36	5.22 ± 1.18	9.409	<0.001
DAS28 (points)		6.80 ± 1.20	4.25 ± 0.87	10.978	<0.001
PSQI (points)		14.40 ± 2.01	9.67 ± 1.46	12.140	<0.001
HAQ-DI (points)		2.40 ± 0.75	1.24 ± 0.43	9.318	<0.001

Note: VAS, Visual Analog Scale; DAS28, Rheumatoid arthritis disease activity; PSQI, Pittsburgh Sleep Quality Index; HAQ-DI, Health Assessment Questionnaire-Disability Index; *, the continuity correction chi-square test was used.

### Factors Influencing Depression in Patients with Endometriosis and Rheumatoid Arthritis

Dysmenorrhea, dyspareunia, VAS, DAS28, PSQI, and HAQ-DI are influencing factors of depression in patients with endometriosis and rheumatoid arthritis, as shown in Tables 3,4.

**Table 3.** **Variable assignment standards**.

Variable		Assignment
Dependent variable		
	Group	Observation group = 1, control group = 0
Independent variable		
	Age	Original value
	Educational level	College degree and above = 1, high school and below = 0
	Reproductive history	Not fertilized = 1, already fertilized = 0
	Average sleep time	Original value
	Dysmenorrhea	Yes = 1, no = 0
	Dyspareunia	Yes = 1, no = 0
	Pelvic pain	Yes = 1, no = 0
	VAS	Original value
	DAS28	Original value
	PSQI	Original value
	HAQ-DI	Original value

Note: VAS, Visual Analog Scale; DAS28, Rheumatoid arthritis disease activity; PSQI, Pittsburgh Sleep Quality Index; HAQ-DI, Health Assessment Questionnaire-Disability Index.

**Table 4.** **Binary logistic regression analysis of depression in patients with endometriosis and rheumatoid arthritis**.

Variable	B	S.E.	Wald	*p*-value	OR (95% CI)
Age	1.033	0.827	1.560	0.212	2.809 (0.556–14.204)
Education level	0.665	0.448	2.206	0.137	1.944 (0.809–4.673)
Reproductive history	0.239	0.253	0.894	0.345	1.270 (0.773–2.087)
Average sleep time	−0.250	0.603	0.171	0.679	0.779 (0.239–2.541)
Dysmenorrhea	1.278	0.460	7.711	0.005	3.589 (1.456–8.845)
Dyspareunia	1.086	0.433	6.309	0.012	2.964 (1.270–6.918)
Pelvic pain	−1.074	0.643	2.786	0.095	0.342 (0.097–1.026)
VAS	0.934	0.274	11.620	0.001	2.545 (1.487–4.354)
DAS28	1.342	0.471	8.124	0.004	3.828 (1.521–9.633)
PSQI	1.372	0.471	8.496	0.004	3.942 (1.567–9.914)
HAQ-DI	1.260	0.477	6.995	0.008	3.527 (1.386–8.975)

Note: VAS, Visual Analog Scale; DAS28, Rheumatoid arthritis disease activity; PSQI, Pittsburgh Sleep Quality Index; HAQ-DI, Health Assessment Questionnaire-Disability Index. OR, odds ratio; CI, confidence interval.

## Discussion

This study aimed to investigate the prevalence and influencing factors of depression in patients with endometriosis combined with rheumatoid arthritis. By using three different depression screening tools—DSM-5, HAMD, and SDS—the detection rates of depression were found to be 18.52%, 19.44%, and 16.67%, respectively, with no significant differences among the three methods. This finding indicates that the internationally accepted DSM-5 diagnostic criteria and the HAMD and SDS assessment tools are effectively applicable in detecting depression in this specific patient population. The binary logistic regression analysis further confirmed the significant impact of pain symptoms (dysmenorrhea, dyspareunia, and pelvic pain) and the VAS, DAS28, PSQI, and HAQ-DI scores on the occurrence of depression in patients with endometriosis combined with rheumatoid arthritis.

Compared with previous studies [19, 20], the present research confirmed the association between dysmenorrhea and depression. A meta-analysis by Esther van Barneveld *et al*. [21] found similar results, indicating that patients with endometriosis often experience depressive and anxiety symptoms associated with chronic pain. Dietrich *et al*. [22] suggested that severe primary dysmenorrhea could trigger chronic pain-related psychological symptoms. The mechanism by which dysmenorrhea contributes to depression may involve multiple physiological and psychological factors. Physiologically, pain transmission and the release of inflammatory mediators may activate the central nervous system, affecting mood regulation pathways and thereby increasing the risk of depression [23]. Additionally, the persistent pain state may lead to decreased sleep quality, heightened psychological stress, and further exacerbate or induce depressive symptoms [24].

This study also observed the impact of dyspareunia on depression, consistent with the findings of Facchin F *et al*. [23], both indicating a close link between sexual dysfunction and mental health. The relationship between dyspareunia and depression may be mediated through various pathways. Psychologically, sexual dysfunction may lead to decreased self-esteem and increased psychological stress, thereby promoting the occurrence of depression [25].

Regarding rheumatoid arthritis activity, a significant correlation was found between DAS28 scores and depression, which aligns with the results of Hughes M *et al*. [26] and Kwiatkowska *et al*. [27], who showed a close relationship between disease activity levels and depression in patients with rheumatoid arthritis. The link between rheumatoid arthritis activity and depression can be partially explained by the complex interactions between inflammatory mediators in the nervous and immune systems. An exacerbated inflammatory response may affect neurotransmitter release and neuronal activity in the brain through multiple pathways, leading to changes in mood and cognitive functions, including the occurrence of depression [28, 29].

This study emphasized the negative impact of functional impairment (measured by HAQ-DI scores) on depression. Uda M *et al*. [30] indicated a correlation between HAQ-DI scores and depressive symptoms. A cross-sectional study by Ruhaila and Chong [31] showed a significant positive correlation among depressive symptoms, disease activity, pain, and HAQ scores. The relationship between functional impairment and depression may reflect patients’ perceived decline in quality of life and adaptive capacity. Functional impairment can lead to reduced social interactions and decreased self-care ability, thereby increasing the prevalence of depression.

PSQI scores, as an indicator of sleep quality, were shown to be associated with the occurrence of depression. The relationship between poor sleep quality and depression may be a bidirectional process. Chronic diseases, such as endometriosis and rheumatoid arthritis, may cause pain and discomfort, affecting patients’ sleep quality. Poor sleep quality or insufficient sleep may affect the stability of neurotransmitters in the brain, increasing the risk of depression [23]. Thus, a bidirectional influence can be observed between sleep disorders and mood disorders.

The mechanisms behind the observed results involve physiological and psychological pathways. Pain symptoms, such as dysmenorrhea, dyspareunia, and pelvic pain, likely activate the central nervous system through pain transmission and the release of inflammatory mediators, which can affect mood regulation pathways and increase the risk of depression [23]. Additionally, chronic pain may lead to decreased sleep quality, which further exacerbates psychological stress and depressive symptoms [23]. The significant correlation between DAS28 scores and depression in patients with rheumatoid arthritis can be explained by the complex interactions between inflammatory mediators and the nervous and immune systems. Increased inflammation may affect neurotransmitter release and neuronal activity in the brain, leading to mood changes and depression [28, 29]. Functional impairment, as indicated by HAQ-DI scores, likely contributes to depression by reducing patients’ perceived quality of life and their ability to adapt, leading to increased social isolation and decreased self-care ability [30, 31]. Poor sleep quality, as indicated by PSQI scores, may contribute to depression by affecting neurotransmitter stability in the brain, thereby increasing the risk of mood disorders [23].

Despite providing new insights into depression in patients with endometriosis combined with rheumatoid arthritis, this study has several limitations. First, the cross-sectional design precluded the determination of causal relationships. Second, the study sample was drawn from a single center, potentially introducing selection bias and limiting the generalizability of the results. Third, this study did not account for certain potential influencing factors such as patients’ medication regimens and social support. Lastly, the positive sample size reported in this study did not meet the required sample size, which could affect the robustness of the results. However, based on the odds ratio (OR) values, confidence intervals, and goodness-of-fit of the binary logistic regression model, the modeling was successful, although the results should be interpreted with caution. Future research should adopt multicenter, large-sample, longitudinal designs combining biological markers and psychological assessment tools to further explore the mechanisms of depression in this specific population and validate additional influencing factors and intervention strategies.

Despite the aforementioned limitations, this study revealed a high prevalence of depression in patients with endometriosis combined with rheumatoid arthritis and preliminarily explored its influencing factors. This study also provides guidance on the selection of depression measurement tools for this patient group. The findings offer clinicians a basis for identifying and intervening in the psychological health issues of these patients. Early diagnosis and treatment of depression can significantly improve patients’ quality of life and treatment outcomes.

## Conclusion

Patients with endometriosis and rheumatoid arthritis are at high risk of depression. The internationally accepted diagnostic criteria for depression, DSM-5, can accurately detect the depression status of patients with endometriosis and rheumatoid arthritis. Some easier-to-operate depression assessment tools, such as HAMD and SDS, showed good results in detecting the depression status of these patients. They can be used as depression assessment tools in clinical practice. In addition, dysmenorrhea, dyspareunia, VAS, DAS28, PSQI, and HAQ-DI are influencing factors for depression in patients with endometriosis and rheumatoid arthritis, and they can provide reference for the clinical diagnosis and treatment of depression.

## Availability of Data and Materials

The datasets for this study are available from the corresponding author on reasonable request.

## References

[1] Horne AW, Missmer SA (2022). Pathophysiology, diagnosis, and management of endometriosis. *BMJ (Clinical Research Ed.)*.

[2] Koninckx PR, Fernandes R, Ussia A, Schindler L, Wattiez A, Al-Suwaidi S (2021). Pathogenesis Based Diagnosis and Treatment of Endometriosis. *Frontiers in Endocrinology*.

[3] Taylor HS, Kotlyar AM, Flores VA (2021). Endometriosis is a chronic systemic disease: clinical challenges and novel innovations. *Lancet (London, England)*.

[4] Amro B, Ramirez Aristondo ME, Alsuwaidi S, Almaamari B, Hakim Z, Tahlak M (2022). New Understanding of Diagnosis, Treatment and Prevention of Endometriosis. *International Journal of Environmental Research and Public Health*.

[5] Filip L, Duică F, Prădatu A, Crețoiu D, Suciu N, Crețoiu SM (2020). Endometriosis Associated Infertility: A Critical Review and Analysis on Etiopathogenesis and Therapeutic Approaches. *Medicina (Kaunas, Lithuania)*.

[6] Warzecha D, Szymusik I, Wielgos M, Pietrzak B (2020). The Impact of Endometriosis on the Quality of Life and the Incidence of Depression-A Cohort Study. *International Journal of Environmental Research and Public Health*.

[7] Škegro B, Bjedov S, Mikuš M, Mustač F, Lešin J, Matijević V (2021). Endometriosis, Pain and Mental Health. *Psychiatria Danubina*.

[8] Yang YT, Jiang XY, Xu HL, Chen G, Wang SL, Zhang HP (2023). Autoimmune Disease-Related Hub Genes are Potential Biomarkers and Associated with Immune Microenvironment in Endometriosis. *International Journal of General Medicine*.

[9] Tańska K, Gietka-Czernel M, Glinicki P, Kozakowski J (2023). Thyroid autoimmunity and its negative impact on female fertility and maternal pregnancy outcomes. *Frontiers in Endocrinology*.

[10] Adewuyi EO, Mehta D, Nyholt DR, International Endogene Consortium (IEC), 23andMe Research Team (2022). Genetic overlap analysis of endometriosis and asthma identifies shared loci implicating sex hormones and thyroid signalling pathways. *Human Reproduction (Oxford, England)*.

[11] Shafrir AL, Palmor MC, Fourquet J, DiVasta AD, Farland LV, Vitonis AF (2021). Co-occurrence of immune-mediated conditions and endometriosis among adolescents and adult women. *American Journal of Reproductive Immunology (New York, N.Y.: 1989)*.

[12] Chen SF, Yang YC, Hsu CY, Shen YC (2021). Risk of Rheumatoid Arthritis in Patients with Endometriosis: A Nationwide Population-Based Cohort Study. *Journal of Women’s Health (2002)*.

[13] Pham HT, Vu-Thi H, Le CT, Dau QL, Nguyen MD, Nguyen-Van H (2024). Characteristics of depressive disorders in patients with rheumatoid arthritis and some related factors. *European Review for Medical and Pharmacological Sciences*.

[14] Szypłowska M, Tarkowski R, Kułak K (2023). The impact of endometriosis on depressive and anxiety symptoms and quality of life: a systematic review. *Frontiers in Public Health*.

[15] Parker G, Malhi GS (2019). Persistent Depression: Should Such a DSM-5 Diagnostic Category Persist? Canadian Journal of Psychiatry. *Revue Canadienne De Psychiatrie*.

[16] Ramos-Brieva JA, Cordero Villafáfila A (1986). Relation between the validity and reliability of the Castillian version of the Hamilton Rating Scale for Depression. *Actas Luso-espanolas De Neurologia, Psiquiatria Y Ciencias Afines*.

[17] Sonnby K, Skordas K, Vadlin S, Olofsdotter S, Nilsson KW, Ramklint M (2022). Psychometric validation of two versions of the adolescent Depression Self-Rating Scale (DSRS-A and DSRS-A Screener). *Nordic Journal of Psychiatry*.

[18] Lee SY, Koo YJ, Lee DH (2021). Classification of endometriosis. *Yeungnam University Journal of Medicine*.

[19] Verma K, Baniya GC (2022). Prevalence of Depression, Anxiety and Quality of Life in Adolescent Girls with Dysmenorrhoea in a Remote Area of Western Rajasthan. *Journal of Obstetrics and Gynaecology of India*.

[20] Zhao S, Wu W, Kang R, Wang X (2021). Significant Increase in Depression in Women with Primary Dysmenorrhea: A Systematic Review and Cumulative Analysis. *Frontiers in Psychiatry*.

[21] van Barneveld E, Manders J, van Osch FHM, van Poll M, Visser L, van Hanegem N (2022). Depression, Anxiety, and Correlating Factors in Endometriosis: A Systematic Review and Meta-Analysis. *Journal of Women’s Health (2002)*.

[22] Dietrich H, Knobel C, Portmann L, Metzler J, Muendane A, Niggli A (2023). Endometriosis features and dienogest tolerability in women with depression: a case-control study. *The European Journal of Contraception & Reproductive Health Care: the Official Journal of the European Society of Contraception*.

[23] Facchin F, Buggio L, Roncella E, Somigliana E, Ottolini F, Dridi D (2021). Sleep disturbances, fatigue and psychological health in women with endometriosis: a matched pair case-control study. *Reproductive Biomedicine Online*.

[24] Haidary M, Arif S, Hossaini D, Madadi S, Akbari E, Rezayee H (2024). Pain-Insomnia-Depression Syndrome: Triangular Relationships, Pathobiological Correlations, Current Treatment Modalities, and Future Direction. *Pain and Therapy*.

[25] Facchin F, Buggio L, Dridi D, Barbara G, Vercellini P (2021). The Subjective Experience of Dyspareunia in Women with Endometriosis: A Systematic Review with Narrative Synthesis of Qualitative Research. *International Journal of Environmental Research and Public Health*.

[26] Hughes M, Chalk A, Sharma P, Dahiya S, Galloway J (2021). A cross-sectional study of sleep and depression in a rheumatoid arthritis population. *Clinical Rheumatology*.

[27] Kwiatkowska B, Kłak A, Maślińska M, Mańczak M, Raciborski F (2018). Factors of depression among patients with rheumatoid arthritis. *Reumatologia*.

[28] Han KM, Ham BJ (2021). How Inflammation Affects the Brain in Depression: A Review of Functional and Structural MRI Studies. *Journal of Clinical Neurology (Seoul, Korea)*.

[29] Richardson B, MacPherson A, Bambico F (2022). Neuroinflammation and neuroprogression in depression: Effects of alternative drug treatments. *Brain, Behavior, & Immunity - Health*.

[30] Uda M, Hashimoto M, Uozumi R, Torii M, Fujii T, Tanaka M (2021). Factors associated with anxiety and depression in rheumatoid arthritis patients: a cross-sectional study. *Advances in Rheumatology (London, England)*.

[31] Ruhaila AR, Chong HC (2018). Self-reported symptoms of depression, anxiety and stress among patients with Rheumatoid Arthritis in a Malaysian rheumatology centre - prevalence and correlates. *The Medical Journal of Malaysia*.

